# Management of Oligometastatic Breast Cancer: An Expert Committee’s Opinion

**DOI:** 10.3390/curroncol30020108

**Published:** 2023-01-19

**Authors:** Dominique Leblanc, Guy Cantin, Alexandra Desnoyers, Jean Dufresne, Giuseppina Laura Masucci, Valérie Panet-Raymond, Éric Poirier, Sara Soldera, Isabelle Gingras

**Affiliations:** 1 Centre Hospitalier Universitaire de Québec—Université Laval, Québec, QC G1V 0A6, Canada; 2 Centre Hospitalier Universitaire de Sherbrooke, Sherbrooke, QC J1H 5N4, Canada; 3 Centre Hospitalier Universitaire de Montréal, Montreal, QC H2X 0C1, Canada; 4 Centre Universitaire de Santé McGill, Montreal, QC H4A 3J1, Canada; 5 Hôpital Charles-Le Moyne, Greenfield Park, QC J4V 2H1, Canada; 6 Hôpital du Sacré-Coeur, Montreal, QC H4J 1C5, Canada

**Keywords:** breast cancer, metastasis, management, expert opinion

## Abstract

Patients with oligometastatic breast cancer (BC) are candidates of choice for metastasis-directed therapy (MDT). This paper summarizes the opinions of an expert committee about the management of oligometastatic BC. The experts could complete the questionnaire from 13 September 2021, to 10 October 2021, followed by a discussion. The experts were physicians working in the Province of Quebec (Canada) and specialized in BC care, including surgical oncologists, medical oncologists, and radiation oncologists. The experts provided their opinions about the context of the disease and therapeutic approach, local and systemic therapies, and the prognosis of oligometastatic BC. In addition to the expert panel’s opinions about the management of oligometastatic disease per se, the experts stated that a prospective data registry should be implemented to collect data about oligometastatic BC to improve knowledge about oligometastatic BC and implement data-driven MDT. These data could also allow for the design of treatment algorithms. In conclusion, this paper presents the expert panel’s opinions about the management of oligometastatic BC and highlights the needs to be met to improve the care of this condition.

## 1. Introduction

Metastatic cancer has been traditionally considered incurable, and its treatment is mainly aimed at extending life, managing symptoms, and maintaining the quality of life [1,2,3]. The concept of the oligometastatic state, described by Hellman and Weichselbaum [4], changed this paradigm. They suggested that metastatic disease is a continuous spectrum, from disease confined to the primary tumor and lymph nodes to widespread metastases [4]. In the intermediate state (i.e., the oligometastatic state), the disease could have a more indolent biology than the widespread metastatic state [5]. Therefore, in this intermediary state, aggressive treatment of all tumors (both the primary and the metastases) could result in a cure. Still, the exact definition of the oligometastatic state varies among studies, with some using up to eight metastases [6], but the most common definition uses up to five metastases [7]. Of note, these criteria are based on imaging and are prone to change according to changes in imaging technologies and guidelines. Recommendations for the diagnosis of oligometastatic disease are available from the European Organization for Research and Treatment of Cancer (EORTC), the European Society for Radiotherapy and Oncology, (ESTRO) [8,9,10], and the American Society for Radiation Oncology together with ESTRO, are working on recommendations. 

Since the concept of oligometastasis was proposed, several high-quality trials of systemic therapy with/without metastasis-directed therapies (MDTs) were performed in various cancers, including colorectal cancer [11], non-small-cell lung cancer [12,13,14], prostate cancer [15,16], and breast cancer (BC) [17]. Importantly, about 50% of the patients with metastatic BC enrolled in phases 2 and 3 clinical trials have one or two metastases [18,19,20,21,22,23]. Such patients might have a better prognosis than patients with widespread metastases. Indeed, early [24] and more recent [25] data showed that having five or fewer metastases was predictive of survival. In such patients, MDT could improve survival. A case series of 467 patients with BC and lung metastases showed improved survival in patients with complete vs. incomplete metastasectomy [26]. A series of patients with metastatic BC showed survival benefits in patients with disease control before liver metastasis resection vs. uncontrolled disease before surgery [27]. Similar results were also observed for brain [28,29,30] and bone [31] metastases. Radiotherapy and interventional radiology are other options for MDT, with highly selective options such as stereotactic body radiation therapy (SBRT) or radiofrequency ablation (RFA) having benefits in patients with lung [32], liver [32], adrenal [33], and multisite [34,35] BC metastases.

Compared with other cancer types, patients with BC have longer survival, especially those with hormone receptor (HR)-positive BC and bone-only metastases [36,37,38]. Therefore, patients with oligometastatic BC are candidates of choice for MDT. Still, despite encouraging results from observational studies and clinical trials [17,26,28,29,30,31,32,33,34,35], vast disparities exist among centers. This paper presents the opinions of an expert committee about oligometastatic BC conducted among BC experts from the Province of Quebec (Canada). The responses to the initial questionnaire were summarized before being discussed to reach the experts’ opinions on the management of oligometastatic BC.

## 2. Materials and Methods

### 2.1. Study Design and Participants

This initial questionnaire was conducted online from September 13, 2021, to October 10, 2021. The participants (the authors of this manuscript) are physicians working in the Province of Quebec (Canada) and specializing in breast cancer care, including surgical oncologists, medical oncologists, and radiation oncologists. All participating experts had at least 7 years of experience managing metastatic cancer in their respective specialties. When building the initial panel, the experts who refused to participate were not included in the panel.

### 2.2. Initial Questionnaire

The initial questionnaire was divided into three parts: (1) disease context and therapeutic approach, (2) local therapies, and (3) prognosis. The specific questions were as follows. The responses were either yes/no (with the possibility of adding comments) or free text. The questions were formulated by the instigating authors based on contemporary clinical issues encountered in routine practice.

The initial questionnaire was web-based through the Within3 web meeting services (Westlake, OH, USA), and the participants could answer at their leisure. The participants could leave an open-text comment for each question.

### 2.3. Data Analysis

The answers to each question were summarized as *n* (%) or as summarized comments.

## 3. Experts’ Opinions and Discussion

### 3.1. Disease Context and Therapeutic Approach

The participants’ answers are provided in Table 1. There was no consensus about whether the definition of oligometastatic disease is the same for all types of primary tumors, with 40% of the experts saying yes and 60% saying no. A participant believed that the definition could be the same, but the pathological characteristics must prevail. The panel suggests that oligometastatic disease should be defined as three–five metastases affecting a limited number of disease sites. Oligometastases can be treated locally. Still, no internationally recognized definition has been suggested [8,9,10]. 

Regarding age, the panel was unanimous that the patient’s age should influence the management of the oligometastatic disease. Indeed, it is the panel’s opinion that the factors influencing the therapeutic decision should be age, comorbidities, disease control, recurrence timing, treatment toxicity, and patient tolerance. An unplanned subgroup analysis of the MF07-01 trial suggested an interaction between young age (<55 years) and the benefit of locoregional treatment followed by systemic therapy for de novo stage IV BC [39]. In patients with colorectal cancer, Onaitis et al. [40] suggested that age <65 years should be considered when discussing the chance of curing solitary lung metastasis. Younger patients will have fewer comorbidities and a higher tolerance for treatments. Szturz et al. [41] suggested that patients >70 years of age should undergo a frailty test and a comprehensive geriatric assessment before treatments are undertaken. The assessment should include functional status, comorbidities, cognition, nutritional status, social support, psychological status, and polypharmacy [42]. Still, what the best age cutoff is in relation to other parameters will have to be determined in future studies.

The therapeutic approach, according to the synchronous/metasynchronous nature of the oligometastases in relation to the primary tumor, should be discussed in tumor boards for each case. A metasynchronous disease provides more information about the biology of the tumor, its aggressiveness, and its response to treatments. On the other hand, a synchronous disease does not provide information about aggressiveness. Since a synchronous disease is usually a new diagnosis, a systemic treatment is usually given first. Nevertheless, a participant suggested that “all-in” aggressive management can be appropriate for a synchronous disease in selected patients and that the treatment plan should begin with systemic therapy. A study in lung cancer suggested that the radical treatment of oligometastases was equally effective for synchronous and metachronous disease [43]. Patients with synchronous oligometastatic BC might have better outcomes than patients with metachronous BC [44,45], possibly because patients with metachronous BC often were exposed to adjuvant treatments that might have selected a resistant clone that caused the metastasis. Supporting this observation, patients with previously untreated metasynchronous BC have similar outcomes compared with those with synchronous BC [44,45]. Therefore, the main issue is whether a metachronous disease is a real metachronous disease or a synchronous disease in which the metastases are simply too small to be detected by the available imaging modalities [46].

The experts are unanimous regarding whether treatment should be selected irrespective of the oligometastasis location. The biology of oligometastases at different sites is different, and the treatment should be different, too. Treatment should also be selected according to the histological subtypes and their known sensitivity to systemic agents. Still, the available data are limited. Nevertheless, studies suggested improved outcomes after the radical treatment of lung [26,47], liver [27,48], adrenal [49], bone [31], and brain [28,29,30] metastases, suggesting that the location of the metastasis does not influence the outcomes of radical treatment. Similar outcomes were observed after radiotherapy for lung [32], liver [50], adrenal [33], and multi-site [6,34] metastases.

The experts were also unanimous that not all oligometastatic BCs can be treated with curative intent. The size of the metastasis, its location, its aggressiveness, as well as the patient’s comorbidities, and performance status, can all influence the decision for metastasectomy. Oligometastatic BC can be impossible to treat curatively if resection is anatomically impossible or the secondary effects are too important. In such cases, the patients will have to receive palliative-intent treatments [51,52,53].

### 3.2. Local Therapies

The participants’ answers are provided in Table 2. The panel believes that young patients, patients with limited disease, patients without serious comorbidities, and patients with a good response to systemic therapy should undergo metastasectomy when possible. According to the panel, metastasectomy increases survival chances, as shown by previous studies [15,26,27,41,47,54], but data are limited. Still, as discussed above, the exact indications and selection criteria for metastasectomy remain to be determined.

The panel considers that metastasectomy should be performed after systemic therapy, as supported by available clinical trials [39,55,56,57]. On the other hand, the timing of radiotherapy would depend on the type of treatment and the expected outcome. The timing can be either concomitant or sequential. Studies of concomitant radiotherapy and CDK4/6 reported relatively favorable outcomes like pain relief and disease stabilization [58,59,60,61,62]. There is no evidence for the timing of radiotherapy in relation to immunotherapy [63]. Radiotherapy without concomitant therapy is known to be effective on metastases, but the exact timing of radiotherapy in relation to other treatments is poorly known since available studies included patients with a wide variety of different timings [17,34,52,54,55]. Still, the NRG-BR002 trial, presented at ASCO 2022 but not yet published, suggested that adding MDT (radiotherapy or surgery) to systemic therapy did not improve survival [64]. Of note, NRG-BR002 was a phase IIR trial, and the sample size was small; furthermore, only 2% of the patients underwent metastasectomy. It is the panel’s opinion that additional studies on metastasectomy are necessary before concluding.

Cryotherapy and radiofrequency ablation are effective for patients with oligometastatic BC [54,65,66]. Thermal ablation techniques can be used in patients who are ineligible for surgery because of the localization of the lesions or if they are not fit for surgery. Unfortunately, access to these modalities is inconsistent among centers in the province of Quebec. Efforts should be taken to improve the availability of thermal ablation.

### 3.3. Prognosis

The participants’ answers are shown in Table 3. All experts agreed that systemic therapy should be given first, followed by curative treatment if the disease remains stable. Doing so provides a better idea of the aggressiveness of the disease and chemosensitivity, will treat the micrometastases that are not visible at imaging, and will provide treatment to the patient while waiting for special techniques or surgical availability. In the ECOG-ACRIN-E 2018 trial, patients with de novo metastatic BC who had stable disease after 4–8 months of systemic therapy were randomized to pursuing systemic therapy vs. locoregional radical resection of the primary tumor; there were no improvements in survival when treating the primary tumor and regional lymph nodes [67]. On the other hand, a retrospective study showed an impressive improvement in overall survival when the patients underwent surgery once the disease was stabilized with systemic therapy, compared with patients with disease progression before surgery (80 vs. 30 months) [27]. Still, data from prospective trials for the timing of systemic therapy and metastasectomy are lacking.

All experts agreed that systemic treatments should be given before and after surgery if an R0 resection is achieved. Still, whether the systemic treatments should be given only for the usual duration of adjuvant systemic therapy or should be given lifelong if well tolerated is unknown; 17% of the experts thought that treatments could be stopped after 1 year if an R0 resection was performed and if the patient remains disease-free, but that the decision should also be based on the biology of the tumor, the number and location of the resected metastases, treatment tolerance, and the patient’s will. 

It is suggested that the patients treated for an oligometastatic BC be followed up every 3 months using CT, with PET and bone scans every 6 months for the first 2 years. After a few years of stability, the examinations can be performed at longer intervals. Those recommendations are based on the experts’ experience since no consensus or guidelines have been published.

In the presence of a recurrence of the oligometastatic disease, a novel maximal treatment should be undertaken after determining the recurrence biology by biopsy. The same treatments can be tried again if they were stopped and there was no progression for at least 1 year. Again, there are no consensuses or guidelines on the subject.

Circulating tumor DNA (ctDNA) appears to be a novel and promising biomarker for guiding clinical practice [68]. ctDNA can be obtained from a liquid biopsy when a traditional biopsy is impossible and can provide interesting information about tumor evolution, treatment resistance, and heterogeneity during treatments [68,69]. ctDNA can be detected in 86% of patients with metastatic BC [70]. Changes in ctDNA levels can indicate the response to treatments [71,72], and there is an increase in ctDNA levels several months before radiological evidence of progression [72]. In addition, ctDNA can provide data about clonal heterogeneity and potential treatment selection [68,71]. Nevertheless, it is the experts’ opinion that the evidence about ctDNA-based management of oligometastatic BC is still not mature enough to introduce ctDNA into the routine management of such patients. So far, no guidelines have been issued regarding the use of ctDNA in BC.

### 3.4. Recommendations for the near Future

The experts recommend that large-scale data on patients with oligometastatic breast cancer should be collected retrospectively and prospectively at the provincial and national levels. Such data would provide evidence for clinical practice. Management algorithms could also be designed to standardize the management of patients with oligometastatic BC. A large-scale phase III randomized trial would be ideal for providing stronger evidence but would be almost impossible to achieve in real life. Still, ongoing trials might provide some answers, such as STEREO-SEIN (NCT02089100), OLIGOMA (NCT04495309), and CLEAR (NCT03750396). For this reason, the panel thinks a prospective national registry would be the best method to provide evidence. 

## 4. Conclusions

This paper presents the expert panel’s opinions about managing oligometastatic disease in BC. Much work is still necessary to improve and optimize the management of patients with oligometastatic BC. Systemic therapy is the mainstay in treating this condition, and research is still needed to optimize patient care and allow optimal use of localized interventions such as surgery and interventional radiology/radiotherapy. Robust evidence from well-designed clinical trials is necessary to improve the management of oligometastatic BC.

## Figures and Tables

**Table 1:** Summary of the questions and answers for disease context and therapeutic approach.

Questions	Answers
Is the definition of oligometastatic disease the same for all types of primary tumors?	Yes: 40% No: 60%
Does the patient’s age influence the management of the oligometastatic disease?	Yes: 100%
Should the therapeutic approach be the same whether the oligometastatic disease is synchronous or metachronous with respect to the primary disease?	- Each case should be discussed on tumor boards. - A metachronous disease provides more information on the tumor’s biology and response to treatments. - There is often a lack of information about a synchronous disease, delaying curative treatments. A systemic treatment is often the best option.
Should oligometastatic breast cancer management be the same irrespective of the metastatic sites?	No: 100%
Are there situations in which an oligometastatic disease cannot be treated with curative intent?	Yes: 100%

**Table 2:** Summary of the questions and answers for local therapies.

Questions	Answers
Which patients who received systemic treatments for a given period of time without progression should undergo surgery?	- Young patients - Patients with limited disease. - No serious comorbidities. - Good response to systemic treatments.
What is the optimal timing for a metastasectomy?	- After the treatments with a general action.
What is the optimal timing for radiotherapy and systemic treatments?	- It depends on the treatment type and the desired result. - It can be concomitant or sequential. - Chemotherapy and CDK4/6 inhibitors are paused, but not hormonal therapy or trastuzumab.
What is the optimal timing for oligometastasis cryoablation or radiofrequency ablation?	- Patients’ ineligible to surgery. - Surgery is impossible or too risky. - If expertise and equipment are available.

**Table 3:** Summary of the questions and answers for prognosis.

Questions	Answers
Should we first give systemic therapy and then perform a definitive treatment if the disease remains stable?	Yes: 100%
Should we consider systemic treatments before and after surgery if there is an R0 resection?	Yes: 100%
Should we allow the patients to stop systemic treatments after 1 year if there are no signs of recurrence or progression, or if an R0 resection was performed?	Yes: 16.7% No: 83.3%
What is the optimal follow-up for patients with oligometastatic breast cancer?	- Follow-up every 3 months. - CT every 3 months for the first 2 years. - PET and bone scans every 6 months for the first 2 years. - After a few years with a stable disease, the follow-up intervals can be longer.
What should be done in the presence of recurrent oligometastatic lesions?	- New optimal treatment. - Retreatment using a previously effective treatment if it was stopped and no progression was observed for 1 year.
Should circulating tumor DNA be used as a biomarker to guide treatments?	No: 100%
